# Exploring the High-Temperature Stabilization of Cubic Zirconia from Anharmonic Lattice Dynamics

**DOI:** 10.1021/acs.cgd.2c01458

**Published:** 2023-04-13

**Authors:** Kasper Tolborg, Aron Walsh

**Affiliations:** † Department of Materials, Imperial College London, Exhibition Road, London SW7 2AZ, United Kingdom; ‡ Department of Physics, Ewha Womans University, Seoul 03760, Korea

## Abstract

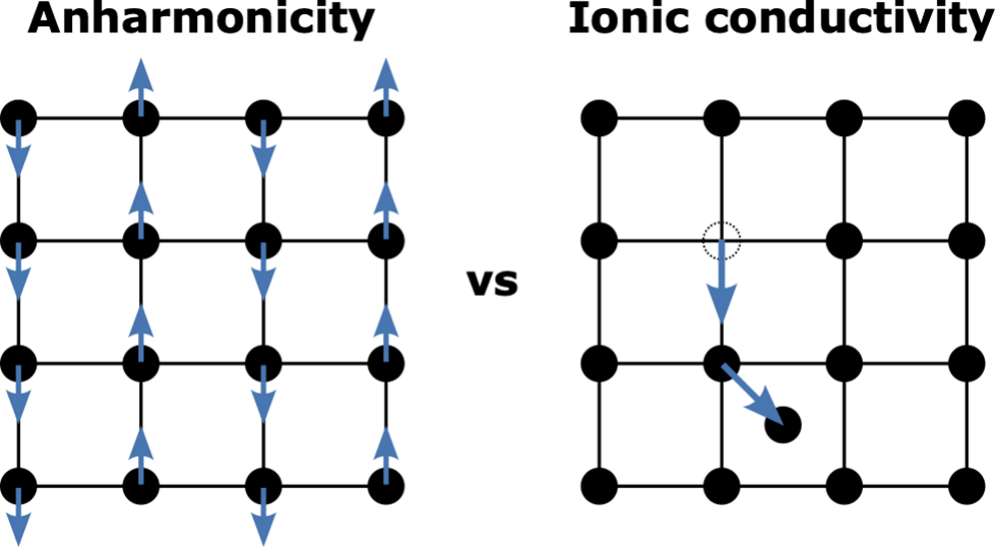

Finite-temperature stability of crystals is of continuous relevance in solid-state chemistry with many important properties only emerging in high-temperature polymorphs. Currently, the discovery of new phases is largely serendipitous due to a lack of computational methods to predict crystal stability with temperature. Conventional methods use harmonic phonon theory, but this breaks down when imaginary phonon modes are present. Anharmonic phonon methods are required to describe dynamically stabilized phases. We investigate the high-temperature tetragonal-to-cubic phase transition of ZrO_2_ based on first-principles anharmonic lattice dynamics and molecular dynamics simulations as an archetypical example of a phase transition involving a soft phonon mode. Anharmonic lattice dynamics calculations and free energy analysis suggest that the stability of cubic zirconia cannot be attributed solely to anharmonic stabilization and is thus absent for the pristine crystal. Instead, an additional entropic stabilization is suggested to arise from spontaneous defect formation, which is also responsible for superionic conductivity at elevated temperatures.

## Abstract 2

Finite-temperature stability of crystals is of continuous importance in solid-state chemistry with many properties only emerging in high-temperature polymorphs. Here, we investigate the high-temperature tetragonal-to-cubic phase transition in zirconia based on first-principles anharmonic lattice dynamics and molecular dynamics simulations. Anharmonic lattice dynamics suggest that the stability of cubic zirconia cannot be attributed solely to vibrational entropic stabilization. Instead, an additional entropic stabilization is suggested to arise from spontaneous formation of Frenkel defect pairs.

## I Introduction

**Figure 1:**
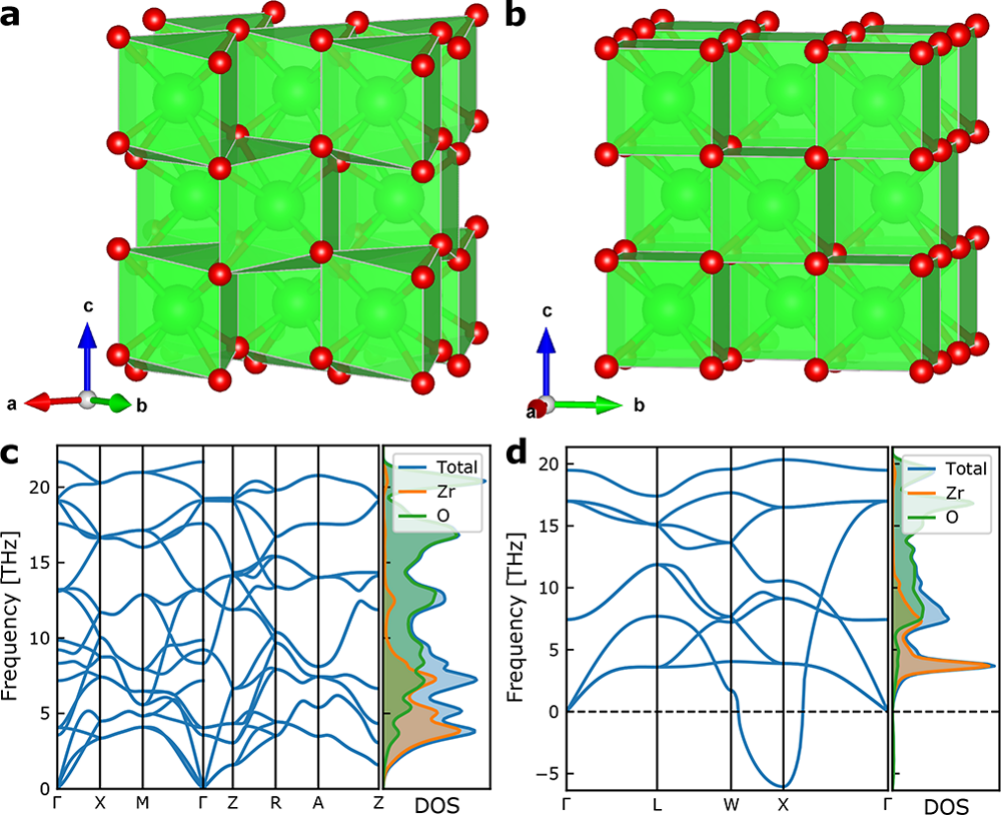
**a** and **b**: crystal structures. **c** and **d**: harmonic phonon dispersions and density of states (DOS) for tetragonal (**a** and **c**) and cubic (**b** and **d**) polymorphs of zirconia. Zr atoms are shown in green, and oxygen atoms in red.

Zirconia, ZrO_2_, is one of the most studied metal oxide ceramics with several interesting properties in both pure and doped forms, which includes high hardness, ionic conductivity, and low thermal conductivity. Thus, it finds application as a hard ceramic,^1,2^ as an electrolyte in solid oxide fuel cells,^3^ and for thermal barrier coatings.^4^ In its pure form, zirconia is observed to have three stable phases at ambient pressure depending on the temperature. At low temperature the structure is monoclinic, at intermediate temperatures a tetragonal polymorph is stable, and at high temperatures the cubic fluorite structure is stable.^5^ The tetragonal and cubic polymorphs are shown in Figure 1 and the monoclinic polymorph in Figure S1.

The phase transition from 7-fold coordinated Zr in the monoclinic polymorph to 8-fold coordinated Zr in the tetragonal polymorph is understood to be a reconstructive phase transition of first-order.^5,6^ In contrast, the transition between tetragonal and cubic polymorphs is thought to be a displacive phase transition of second-order, which is allowed by the group–subgroup relation between the phases.^7^ However, a latent heat has been measured for this transition, suggesting that weak first-order behavior could be present.^8^ Furthermore, there are conflicting reports from computational studies with some suggesting it to be second-order^9,10^ and others suggesting it to be first-order.^11^

Ultimately, the stability of a given phase is determined from its free energy, Δ*G*, in relation to its competing phases.^12,13^ Stability in the solid state is often determined from the internal energy or enthalpy based on density functional theory (DFT) calculations, but when comparing phase stability at different temperatures, entropic contributions must be taken into account.
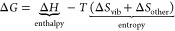
1The main source of entropy in an crystalline solid is usually of vibrational origin, which can to a first approximation be determined using the (quasi-)harmonic approximation to describe the vibrational degrees of freedom. However, when dynamic instabilities are present in the phonon dispersion, which is often the case for high temperature phases, the harmonic approximation breaks down and the vibrational entropy becomes ill-defined.^14^ In such cases, we must resort to an anharmonic treatment of the phonons. In recent years, significant progress has been made in the modeling of anharmonic lattice vibrations including the temperature dependent effective potential method,^15,16^ stochastic self-consistent harmonic approximation,^17,18^ and self-consistent phonon theory.^19,20^ This has paved the way for modeling of finite temperature phonon dispersions, lattice thermal conductivities, as well as free energy calculations beyond the harmonic approximation.^13,16,21^

## II Computational Methods

### II.A Density Functional Theory and Harmonic Phonon Calculations

Density functional theory (DFT) calculations are performed within the projector augmented wave method implemented in the Vienna ab initio simulation package (VASP) employing the PBEsol functional.^22−24^ For zirconia, the PBEsol functional has previously been shown to yield very similar geometries and energy differences to the hybrid functional, HSE06.^25^ Calculations employ a plane wave energy cutoff of 700 eV. Gamma-centered *k*-meshes of 6 × 6 × 4 and 6 × 6 × 6 were used for tetragonal and cubic ZrO_2_, respectively. Harmonic phonon calculations were performed using the finite displacement method in PHONOPY with forces calculated using VASP.^26^ 3 × 3 × 2 and 2 × 2 × 2 conventional cubic supercells were used for tetragonal and cubic zirconia, respectively.

### II.B Self-Consistent Phonon Calculations

Self-consistent phonon calculations were performed with ALAMODE.^27^ 3 × 3 × 2 and 4 × 4 × 4 primitive cells were used for tetragonal and cubic zirconia, respectively. Force constants were determined using compressive sensing. First, a short (2 ps) ab initio molecular dynamics simulation was performed with VASP at a temperature of 1000 K and a step size of 2 fs. 50 equidistant configurations were extracted, and a random displacement of 0.1 Å was added to each atom to avoid strong correlations between configurations, and DFT calculations were performed on each of these configurations. Force constants were fitted using least absolute shrinkage and selection operator (LASSO) regression,^28^ for which the regularization parameter was determined using 10-fold cross-validation.

The self-consistent phonon equations were solved on an 8 × 8 × 8 reciprocal space grid,^19^ and three different additional bubble corrections termed QP[0], QP[S], and QP-NL were included besides the first-order SC1 theory only based on the loop diagram.^29^ Anharmonic free energies were calculated including anharmonic correction from both loop and bubble diagram according to the method of Oba et al.^21^

Effects of volume expansion on the phonon dispersions of cubic zirconia were considered using the method of Oba et al.^21^ Explicit anharmonic phonon calculations were performed at seven different volumes, and the phonon dispersions were interpolated to the minimum free energy volume for each temperature.

### II.C Ab Initio Molecular Dynamics Simulations

Ab initio molecular dynamics (AIMD) simulations of cubic zirconia were performed with VASP. In all cases a time step of 2 fs and a Nosé thermostat were used in an NVT ensemble. For standard AIMD simulations, a plane wave energy cutoff of 700 eV, a 4 × 4 × 4 primitive supercell, and Γ-point sampling was used with a total simulation time of 16 ps. For the constrained MD simulations, oxygen atoms were displaced along the eigenvector of the imaginary phonon mode at the X-point, and one coordinate for each oxygen was kept fixed. These simulations were performed with a plane wave energy cutoff of 400 eV, a 2 × 2 × 2 conventional supercell, and 2 × 2 × 2 k-point sampling, and a total simulation time of 16 ps for each of the 8 points along the X-mode was used. The free energy gradient was extracted using the Blue Moon ensemble as implemented in VASP using the LBLUEOUT tag, and the free energy profile was determined through numerical integration.^30^

## III Results and Discussion

### III.A Anharmonic Lattice Dynamics of ZrO_2_

The monoclinic-to-tetragonal phase transition in zirconia is well-understood within the (quasi-)harmonic approximation, with the tetragonal phase showing a larger vibrational entropy compensating the lower internal energy of the monoclinic phase.^31^ For improved quantitative agreement of the phase transition temperature, anharmonic effects have been included.^10^

From the harmonic phonons of ZrO_2_ in Figure 1, we observe that cubic zirconia is predicted to be dynamically unstable with an imaginary phonon mode at the X-point of the Brillouin zone. The eigenvector of this mode corresponds to a distortion to the tetragonal phase with a doubling of the unit cell. This distortion from the cubic phase is favorable in terms of internal energy. Thus, to understand the observed dynamic stability of this polymorph, we must resort to an anharmonic description, which we perform within the framework of self-consistent phonon (SCPH) theory.^19,20^ The anharmonic force constants are determined using compressive sensing, which allows for extraction of high-order force constants at a significantly lower computational cost compared with direct determination of the force constants using symmetry-adapted finite displacements.^28^

In order to completely describe the relative phase stability of two phases, their free energies must be calculated according to eq 1. However, when two phases are related by a group–subgroup relation, it is common to investigate phase stability as the dynamical stabilization with respect to temperature of the high temperature phase.^19,29,32,33^ In this case, the phase transition temperature is then given as the temperature at which the relevant phonon frequency changes from real to imaginary. Initially, we will follow this approach to investigate the dynamic, local stability of cubic zirconia, but as a complete theory must also be able to predict the correct phase stability in terms of relative free energies, we will take this approach at the end of the section, revealing that description of dynamical stability may not show the full picture in terms of stability of competing phases.

**Figure 2:**
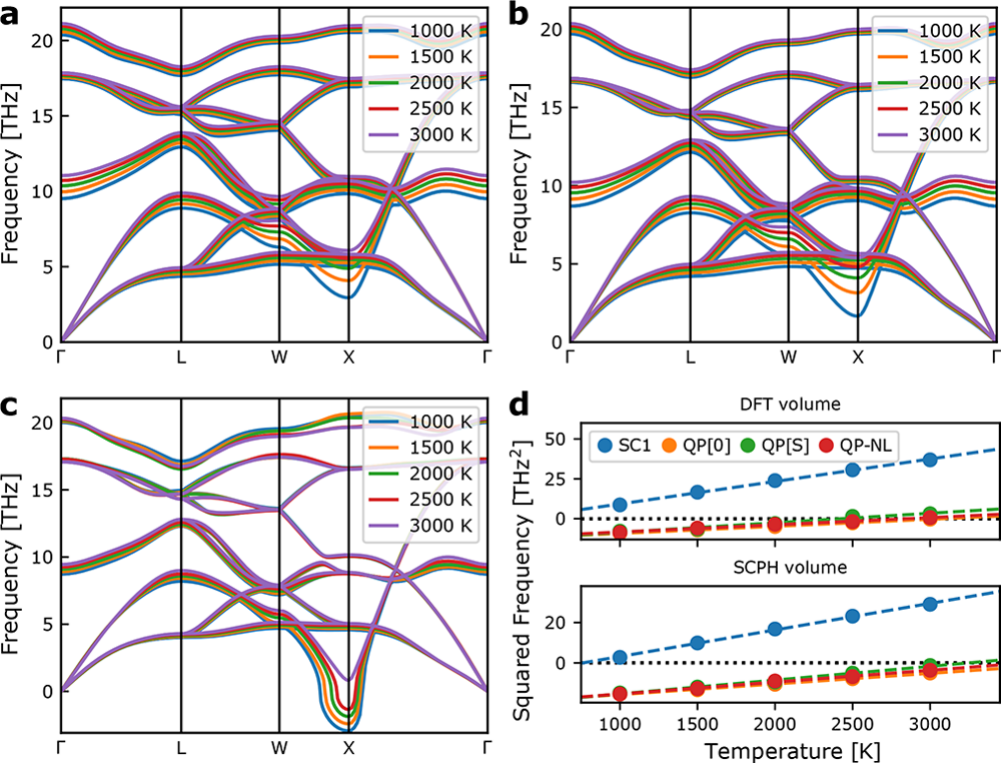
Anharmonic phonon dispersions in cubic zirconia. **a**: SC1 theory at the 0 K DFT volume. **b**: SC1 theory including volume expansion at the SCPH level. **c**: QP-NL phonon theory at the 0 K DFT volume. **d**: temperature dependence of the soft-mode at the X-point for the different levels of theory and at the 0 K DFT volume and including volume expansion, respectively.

At the simplest level of SCPH theory (SC1), corresponding to the first-order expansion of the anharmonic free energy, the fourth-order force constants are included in the self-consistent determination of phonon frequencies via the loop-diagram.^20^ The resulting anharmonic phonon dispersion for cubic ZrO_2_ clearly shows that the material is now predicted to be dynamically stable with no imaginary phonon modes (Figure 2a). However, it is also noted that the phase is predicted to remain stable at significantly lower temperatures than observed experimentally. This is a result of only including fourth-order force constants, which can lead to an overstabilization of soft modes.^29^

It is possible to extend SC1 theory by including higher-order corrections to the free energy. We can first include the effect of volume expansion through the use of the anharmonic free energy,^21^ i.e., coupling of the free energy to lattice degrees of freedom. Second, we can include the next order in the free energy expansion—the so-called bubble correction—which is determined from third-order force constants.^29^

As seen from Figure 2b, volume expansion leads to an increased softening of the mode, but the phase transition is still predicted to occur below 1000 K. Rather, inclusion of third-order force constants in phonon quasi-particle (QP) theory does result in the prediction of a soft-mode phase transition at high temperature (Figure 2c). Figure 2d shows the transition temperature predicted with SC1 and various levels of QP theory at both 0 K DFT volumes and volumes predicted from anharmonic free energies. The transition temperature predicted from QP-NL theory at the 0 K DFT volume, ∼2850 K, is in good agreement with the experimental value of 2650 K,^5^ whereas inclusion of thermal expansion increases the predicted transition temperature to above the experimental melting point. A similar behavior was observed by Tadano et al. for CsPbBr_3_ and was attributed to deficiencies in the underlying PBEsol exchange-correlation functional used to calculate the atomic forces.^29^

Thus, anharmonic phonon theory allows for prediction of the cubic-to-tetragonal phase transition upon cooling, though with a significant dependence of the transition temperature on the level of approximation.

**Figure 3:**
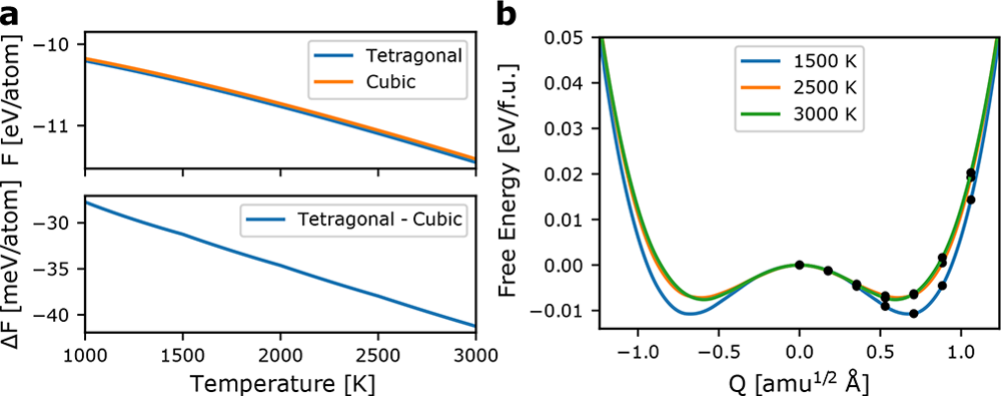
**a**: Free energy and free energy difference of tetragonal and cubic zirconia including anharmonic effects. **b**: Free energy profile from constrained molecular dynamics along the soft-mode at the X-point. The free energy is determined from integration of the free energy gradient in the Blue Moon ensemble, and the black dots indicate the order parameters at which simulations were performed.

As mentioned above, a complete theory should also be able to predict the reverse transition upon heating. In a second-order phase transition, this should manifest itself in a similar phonon mode softening upon increasing temperature, and a convergence of the free energies of the two phases at the phase transition temperature. However, no softening of the phonon modes is observed for the tetragonal polymorph in Figure S5. Furthermore, the tetragonal phase is predicted to be 20–40 meV atom^–1^ more stable at all temperatures when comparing both harmonic and anharmonic free energies (Figures S4, S6, and Figure 3a).

### III.B Entropic Stabilization of Cubic ZrO_2_

Since the anharmonic free energies fail to describe the phase transition, we posit that other entropic factors must stabilize the cubic phase. Given the consistent 20–40 meV atom^–1^ greater stability of the tetragonal phase, an additional entropic stabilization, Δ*S*_other_ in eq 1, on the order of ∼0.01 meV atom^–1^ K^–1^ is needed at the phase transition temperature. We consider three origins: (i) higher-order anharmonic contributions (which are technically another vibrational entropic contribution, Δ*S*_vib_), e.g., the temperature dependent internal coordinates are not included at the present level of theory;^20^ (ii) a dynamic or static ensemble of local tetragonal domains to produce a quasi-cubic phase on average; (iii) fast ionic conductivity, which is a common feature of fluorite type structures.^34,35^ These possibilities can be tested through various aspects of *ab initio* molecular dynamics (AIMD) simulations.

#### 1 Higher-Order Anharmonicity

This contribution is probed using constrained MD with the Blue Moon ensemble along an order parameter given as the collective oxygen displacement along the soft-mode at the X-point.^9,30^ This gives access to the free energy gradient along the order parameter which can be integrated to obtain the free energy as a function of the order parameter. As shown in Figure 3b, the free energy surface remains of double well nature at temperatures up to 3000 K. Thus, no transition is predicted to occur below the melting point. Thus, we cannot attribute the stabilization of cubic zirconia to higher orders of anharmonicity not included with the current level of SCPH. Importantly, with the constrained MD, no ionic diffusion is allowed.

#### 2 Local Tetragonal Domains

The distribution of oxygen atoms around their equilibrium positions from AIMD at 2500 K is shown in Figure S7. Here, no signs of ion off-centering are observed. We do observe strong local correlations corresponding to the displacement of ions along the soft-mode, which exist along all three Cartesian directions, corresponding to the three possible symmetry lowering pathways from cubic to tetragonal. However, this is exactly the expected behavior from a low energy phonon mode. Thus, there are no signs of the cubic phase being an average over multiple locally tetragonal phases. Similar behavior was observed by Carbogno et al.,^25^ who showed that no persistent tetragonal domains were present for their highest simulation temperatures. While there is no evidence to support their formation, we cannot rule out that such domains do emerge over longer length- and/or time scales.

#### 3 Ionic Conductivity

**Figure 4:**
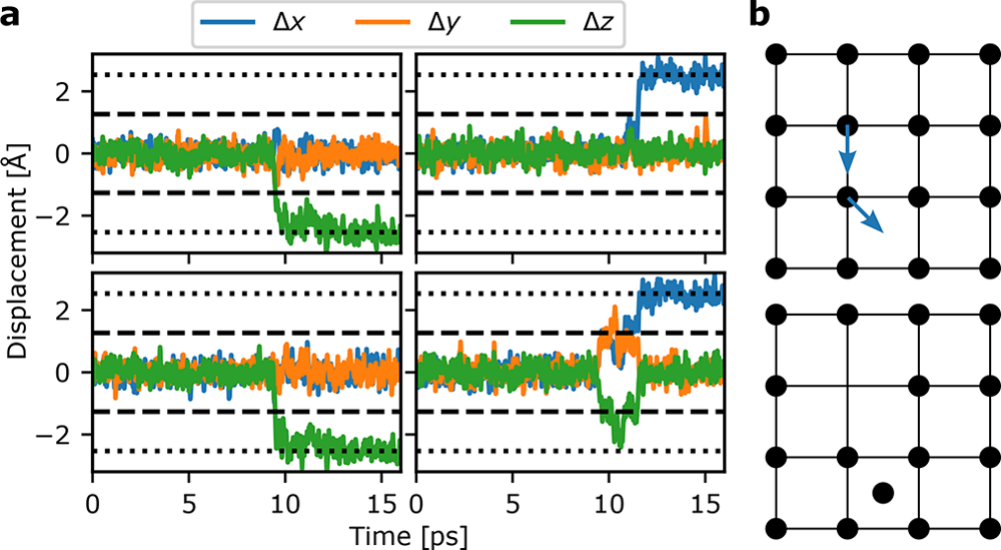
Diffusion in cubic zirconia from AIMD at 2500 K. **a**: Displacements from equilibrium of four selected oxygen atoms as a function of time. The dashed line indicates the displacement that should occur along all three axes to enter the interstitial site, while the dotted line indicates the displacement at should occur along one direction to enter another oxygen site. **b**: Diffusion mechanism showing one oxygen entering an interstitial site immediately followed by another oxygen atom occupying its original position. A Frenkel defect pair is thus created, and an oxygen site is vacant allowing for easy ionic diffusion. The schematic is inspired by ref (35).

Finally, we consider the possibility of stabilization of the cubic fluorite phase due to ionic diffusion. From an AIMD simulation at 2500 K, we observe the spontaneous formation of Frenkel defect pairs consisting of an oxygen vacancy and an interstitial oxygen atom as shown in Figure 4.^36^ This leads to spontaneous diffusion even in a stoichiometric zirconia sample following the mechanism previously proposed for other fluorite type structures including CeO_2_.^35^ In this mechanism, one oxygen atom enters an interstitial site immediately followed by another oxygen atom occupying the empty site. Thus, a Frenkel defect pair is created, and vacancy mediated diffusion can now occur throughout the material.

Besides its importance for ionic transport, the spontaneous defect formation and diffusion must have implications for the phase stability. A simple model for fluorite type structures has been derived by Voronin, in which all interstitial sites are considered accessible and the configurational entropy arising from random occupation of interstitials and vacancies is calculated.^37^ The model is desribed in further detail in Supporting Note II, and the entropy as a function of defect concentration is shown in Figure S8. At the high temperatures considered here (∼2500 K), a defect concentration of only ∼3% would amount to an entropic stabilization on the same order as the free energy difference between cubic and tetragonal phases. Thus, it appears that cubic zirconia could be stabilized by configurational entropy arising from partial melting of the oxygen sublattice through the spontaneous formation of Frenkel defect pairs.

It should be noted that this description only takes into account the configurational entropy in the cubic phase and not a similar entropy in the tetragonal phase, which will also be nonzero. We furthermore note that throughout the present simulation time (16 ps) the defect concentration is less than 3%. Obtaining a converged equilibrium defect concentration is currently beyond the reach of AIMD simulations.

Thus, rather than a purely entropy-driven transition, a likely mechanism is that upon heating, Frenkel defect pairs are created in the tetragonal phase, and since these are created at random positions with random relative orientations, an isotropic “pressure” is exerted upon the crystal resulting in an overall average cubic symmetry. This is similar to the stabilization mechanism in yttria-stabilized-zirconia (YSZ), where the Y substitutions are compensated by oxygen vacancies. Introduction of vacancies has previously been shown to lead to a lowering of the energy difference between cubic and tetragonal zirconia.^25^

## IV Conclusions

In conclusion, we have shown that the cubic-to-tetragonal phase transition in zirconia can be partly described within the framework of anharmonic phonon theory in terms of a mode softening upon cooling in the high-temperature cubic phase. Conventional SC1 theory based only on frequency renormalization from fourth-order force constants fails to describe an adequate softening of the phonon mode responsible for the transition. Inclusion of third-order force constants through the bubble self-energy results in a further mode softening and leads to a prediction of the phase transition temperature in reasonable agreement with experimental observations—though quantitatively highly dependent on the exact details of the quasi-particle correction.

Within anharmonic phonon theory, however, the free energy of the tetragonal phase remains lower than the cubic phase for all temperatures, meaning that the relative stability is not well-described. Thus, it is expected that a further stabilization mechanism is involved. From AIMD simulations, we show that spontaneous formation of Frenkel defect pairs occur in cubic zirconia at elevated temperatures. Thus, we propose that this defect formation is responsible for the stabilization of cubic zirconia through a similar mechanism as that of YSZ. Interestingly, the transition is observed to occur at ∼80% of the melting point temperature, which is similar to the Bredig transition temperature in other fluorite type structures, which is the temperature at which these materials become superionic conductors.^38,39^

Furthermore, a stabilization mechanism involving the creation of defect pairs would explain the weak first-order transition behavior observed experimentally as an enthalpy of transition,^8^ since defect creation requires energy. This suggests that the transition into cubic ZrO_2_ should not be considered a simple second-order group–subgroup transition purely driven by the soft phonon mode.

## Supporting Information Available

Supporting note on self-consistent phonon theory, supporting note on entropy from Frenkel defect pairs, figures of monoclinic ZrO_2_ and its harmonic phonon dispersion, figures on ModeMap renormalized harmonic phonon dispersion in cubic ZrO_2_, harmonic free energies, self-consistent phonon dispersion of tetragonal zirconia, anharmonic free energies of cubic and tetragonal zirconia, histogram of oxygen displacements during AIMD simulations, estimation of entropic contribution from Frenkel defect pair formation (PDF)
