# Supplementary material for: Exploring the High-Temperature Stabilization of Cubic Zirconia from Anharmonic Lattice Dynamics
Source: Cryst Growth Des. 2023 Apr 13;23(5):3314–9. doi: 10.1021/acs.cgd.2c01458 (PMC10161191; doi:10.1021/acs.cgd.2c01458)
Supplement: Supplementary file 1 — cg2c01458_si_001.pdf [file cg2c01458_si_001.pdf]

Supporting information for: Exploring the High-Temperature Stabilisation of Cubic Zirconia from Anharmonic Lattice Dynamics

Kasper Tolborg[1, *] and Aron Walsh[1, 2, †]
[1] *Department of Materials, Imperial College London, Exhibition Road, London SW7 2AZ, United Kingdom*
[2] *Department of Physics, Ewha Womans University, Seoul 03760, Korea*
(Dated: March 30, 2023)

SUPPORTING NOTE I: SELF-CONSISTENT PHONON THEORY

Anharmonic contributions to the lattice dynamics are treated within self-consistent phonon (SCPH) theory in the present article. For a broad and formal introduction to the theory and its implementation in ALAMODE, we refer the reader to the original publications [1–4]. However, in order to ease the reading of this article, we here give a brief introduction to the topic.

Lattice dynamics is based dynamics of ions on the potential energy surface, which we describe with a Taylor expansion as

$$U = U_0 + U_2 + U_3 + U_4 + \cdots , \tag{1}$$

where U_n is the n'th order term in the expansion. These are determined from the force constants, Φ, at n'th order

$$U_n = \frac{1}{n!} \sum \Phi_{\mu_1 \dots \mu_n}(l_1\kappa_1; \dots ; l_n\kappa_n) u_{\mu_1}(l_1\kappa_1) \cdots u_{\mu_n}(l_n\kappa_n), \tag{2}$$

where $u_\mu(l\kappa)$ is the displacement of atom κ in cell l along the cartesian direction μ.

In the harmonic approximation, only the U_2 contribution is retained, and the solution is a set of eigenfrequencies and eigenvector as a function of the reciprocal vector, and band index, j.

Within SCPH theory, anharmonic phonon frequencies can be obtained through the Green's function, $G_{\mathbf{q}jj'}$, by solving the Dyson equation given in matrix form as

$$\left[\mathbf{G}_\mathbf{q}(\omega)\right]^{-1} = \left[\mathbf{G}^0_\mathbf{q}(\omega)\right]^{-1} - \Sigma_\mathbf{q}(\omega), \tag{3}$$

where $\Sigma_\mathbf{q}(\omega)$ is the anharmonic self-energy. Solving the Dyson equation leads to the following equation, which needs to be solved self-consistently for the anharmonic phonon frequencies, ω

$$\det\left[\omega^2 - \mathbf{V}_\mathbf{q}(\omega) = 0\right] \tag{4}$$

$$V_{\mathbf{q}jj'}(\omega) = \omega^2_{\mathbf{q}j}\delta_{jj'} - (2\omega_{\mathbf{q}j})^{\frac{1}{2}}(2\omega_{\mathbf{q}j'})^{\frac{1}{2}}\Sigma_{\mathbf{q}jj'}(\omega). \tag{5}$$

The SCPH equations can be solved to various levels of theory by systematically including higher order diagrams to the phonon self-energy. The simplest level of theory, here termed SC1 theory, includes only the contributions from the loop-diagram, which is based on fourth-order force constants. At this level of theory, and neglecting off-diagonal components, the SCPH equations can be written in diagonal form as [1]

$$\Omega^2_\mathbf{q} = \omega^2_\mathbf{q} + 2\Omega_\mathbf{q} I_\mathbf{q} \tag{6}$$

---

* k.tolborg@imperial.ac.uk
† a.walsh@imperial.ac.uk

$$I_{\mathbf{q}} = \frac{1}{2}\sum_{\mathbf{q}_1} \frac{\hbar\Phi(\mathbf{q};-\mathbf{q};\mathbf{q}_1;-\mathbf{q}_1)}{4\Omega_{\mathbf{q}}\Omega_{\mathbf{q}_q}}\left[1+2n(\Omega_{\mathbf{q}_1})\right], \tag{7}$$

where n is is the Bose-Einstein distribution giving the population of the phonon mode at the relevant temperature, which is how temperature is introduced into the SCPH equations. This leads to a set of renormalised phonon frequencies Ω, which are all real, even if imaginary harmonic frequencies are present. As all frequencies are real by construction of equations, a phase transition should be predicted by following the change in frequency above the phase transition temperature and extrapolating to zero frequency.

The next level of anharmonicity to include originates from the bubble diagram, which is based on third order force constants [3]. In the current implementation, the fully anharmonic Dyson equation, eq. 3, is approximated as

$$\left[\mathbf{G}_{\mathbf{q}}(\omega)\right]^{-1} \approx \left[\mathbf{G}^S_{\mathbf{q}}(\omega)\right]^{-1} - \Sigma^B_{\mathbf{q}}\left[\mathbf{G}^S, \Phi_3\right](\omega), \tag{8}$$

where $\Sigma^B_{\mathbf{q}}$ is the bubble self-energy, and the self-consistency from the Dyson equation is removed, since the Green's function from the SC1 theory, $\mathbf{G}^S_{\mathbf{q}}$, is close to the full Green's function. This leads to the following self-consistent equations

$$(\Omega^B_{\mathbf{q}})^2 = (\Omega^S_{\mathbf{q}})^2 - 2\Omega^S_{\mathbf{q}}\mathrm{Re}\Sigma^B_{\mathbf{q}}(\omega=\Omega^B_{\mathbf{q}}), \tag{9}$$

where $\Omega^S_{\mathbf{q}}$ are the solutions to the SC1 equations in eq. 6. Solving this non-linear (NL) equation results in phonon quasi-particle (QP) frequencies termed QP-NL. Further approximation can be made by setting ω in the self-energy equal to 0 (QP[0] theory) or to $\Omega^S_{\mathbf{q}}$ (QP[S] theory) as used in Fig. 2 in the main text.

Finally, anharmonic free energies can be calculated within the SCPH framework [4]. The phonon frequencies from SC1 theory are used and the free energy is calculated as a summation over these in a similar way as what is done in the harmonic approximation, but with an additional term to satisfy the thermodynamic relation between entropy and free energy. To improve accuracy, the contribution from the bubble diagram is added as an additional term through the bubble self-energy as described above.

SUPPORTING NOTE II: ENTROPY FROM FRENKEL DEFECT PAIRS

A simple model for the configurational entropy of Frenkel defects in fluorite type structures has been derived by Voronin [5]. In this model, all interstitial sites are considered accessible and the configurational entropy from random occupation of interstitials and vacancies is determined. The configurational entropy (per atom) is given as

$$S_{\mathrm{config}} = \frac{k_{\mathrm{B}}}{3}\left[2\ln 2 - (2-x)\ln(2-x) - (1-x)\ln(1-x) - 2x\ln x\right], \tag{10}$$

where x is the fraction of occupied interstitials.

The entropy as a function of defect concentration is plotted in Fig. S8 showing that a defect concentration of around 3% would result in a similar entropic stabilisation as the energetic difference between cubic and tetragonal phases of zirconia.

SUPPORTING FIGURES

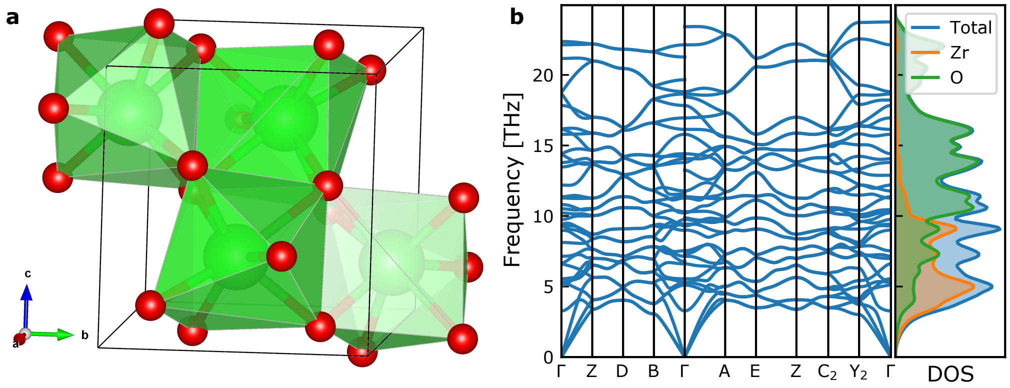

FIG. S1. **a** Crystal structure, and **b** phonon dispersion for monoclinic zirconia.

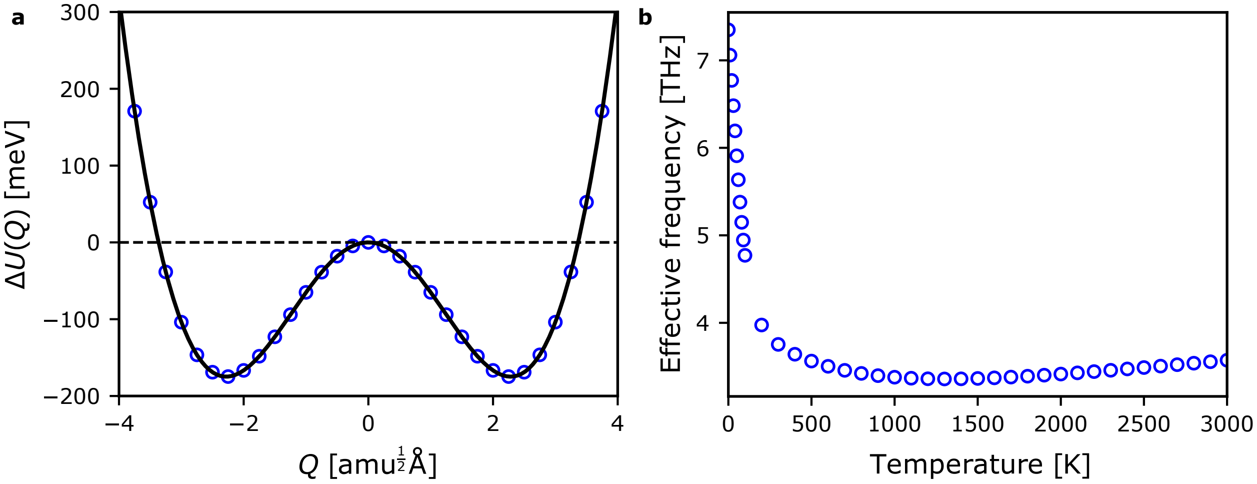

FIG. S2. **a** Potential energy surface of the X-mode in cubic zirconica, and **b** the effective frequency calculated using the method of Skelton et al. [6]

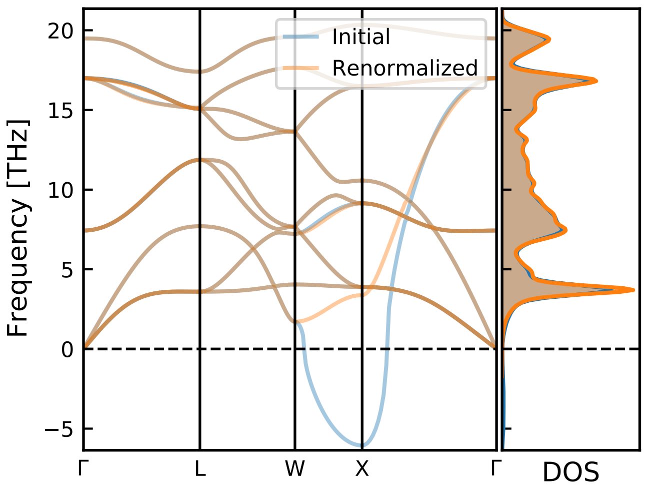

FIG. S3. Conventional and renormalised harmonic phonon dispersion of cubic zirconia using the method of Skelton et al. [6] to obtain only real frequency for calculation of the vibrational free energy. The frequency at 1000 K is used since this is near the flat region of the freqency with temperature and therefore gives a reasonable overall effective frequency for a qualitative comparison of free energies.

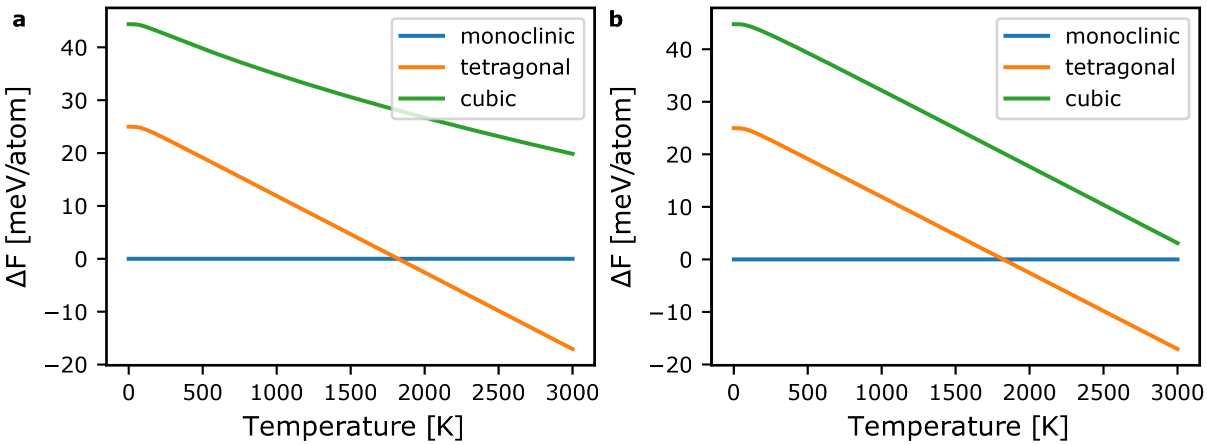

FIG. S4. Free energies including the athermal internal energy and vibrational free energy relative to the monoclinic polymorph as a function of temperature. **a** shows the bare free energies, where imaginary modes are present in the cubic phase, and the free energy is therefore ill-defined, and **b** shows the free energy calculated with the imaginary mode renormalised according to Fig. S3.

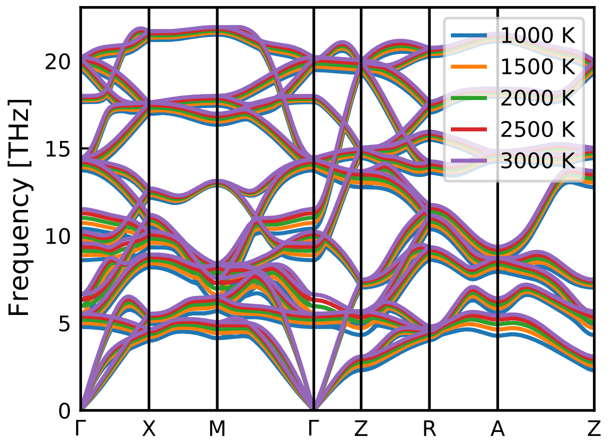

FIG. S5. Temperature dependent phonon dispersion in tetragonal zirconia from self-consistent phonon theory.

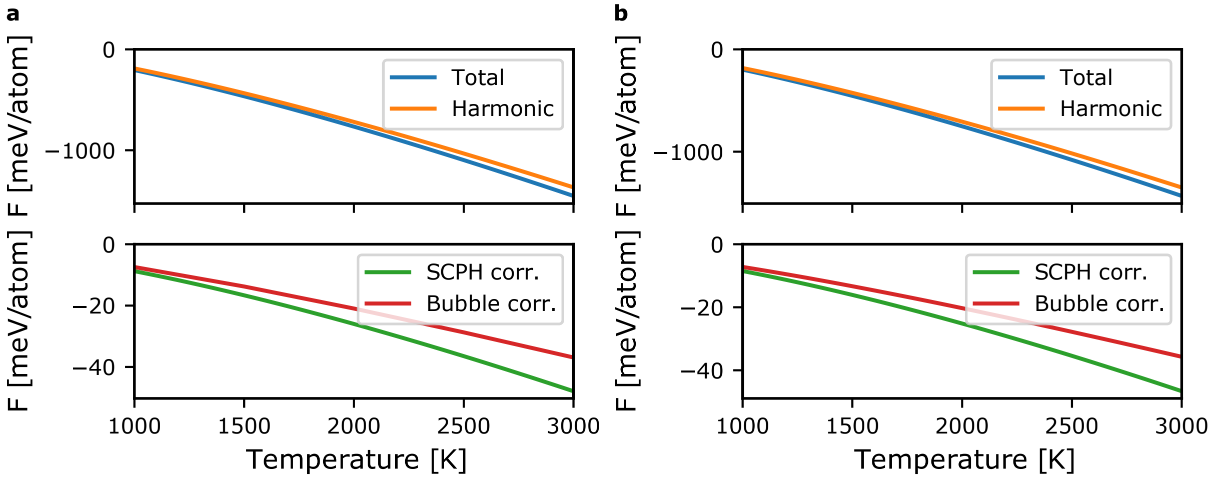

FIG. S6. Anharmonic free energies calculated within self-consistent phonon theory for **a** tetragonal zirconia, and **b** cubic zirconia. For tetragonal zirconia, the bubble correction is only evaluated at 1000, 1500, 2000, 2500 and 3000 K due to its high computational and memory requirements, and interpolated to values in-between.

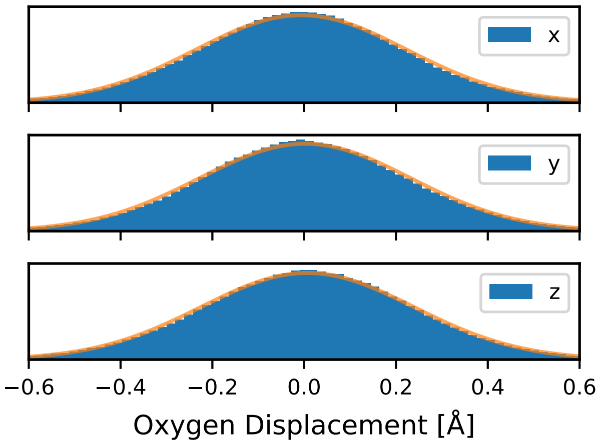

FIG. S7. Histograms of oxygen displacements from their cubic positions along the three cartesian axes from AIMD at 2500 K. The orange lines are Gaussians with the center and width determined from the mean and standard deviation of the oxygen displacements. Diffusing oxygens are excluded from the analysis to probe only the local off-centering of atoms.

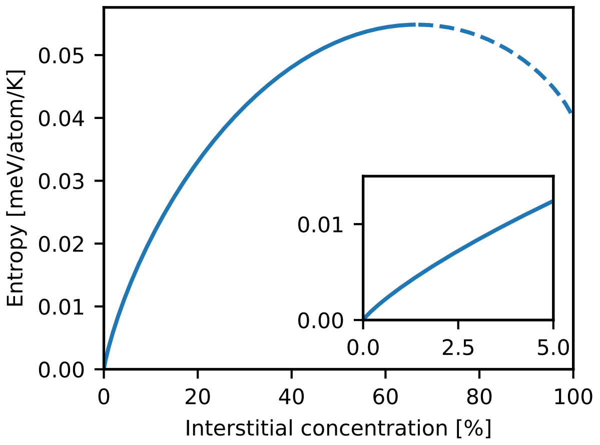

FIG. S8. Entropic contribution from Frenkel defect pairs following a simple configurational model of Voronin [5] given in Eq. (2) in the main text. The interstitial concentration is given as the fraction of occupied interstitials.

[1] T. Tadano and S. Tsuneyuki, Self-consistent phonon calculations of lattice dynamical properties in cubic srtio$_3$ with first-principles anharmonic force constants, Physical Review B **92**, 054301 (2015).
[2] T. Tadano and S. Tsuneyuki, First-principles lattice dynamics method for strongly anharmonic crystals, Journal of the Physical Society of Japan **87**, 041015 (2018).
[3] T. Tadano and W. A. Saidi, First-principles phonon quasiparticle theory applied to a strongly anharmonic halide perovskite, Physical Review Letters **129**, 185901 (2022).
[4] Y. Oba, T. Tadano, R. Akashi, and S. Tsuneyuki, First-principles study of phonon anharmonicity and negative thermal expansion in ScF_3, Physical Review Materials **3**, 033601 (2019).
[5] B. Voronin, Some simple thermodynamic approaches to superionic disorder in fluorite-type crystals: Application to $SrCl_2$ and K_2S, Journal of Physics and Chemistry of Solids **56**, 839 (1995).
[6] J. M. Skelton, L. A. Burton, S. C. Parker, A. Walsh, C.-E. Kim, A. Soon, J. Buckeridge, A. A. Sokol, C. R. A. Catlow, A. Togo, *et al.*, Anharmonicity in the high-temperature c m c m phase of snse: Soft modes and three-phonon interactions, Physical Review letters **117**, 075502 (2016).
